# LncRNA GAS8‐AS1 downregulates lncRNA NEAT1 to inhibit glioblastoma cell proliferation

**DOI:** 10.1002/brb3.2128

**Published:** 2021-05-04

**Authors:** Xiaoming Wu, Tingting Jiang, Rui Huang, Xue Xiao

**Affiliations:** ^1^ Department of Neurosurgery People’s Hospital of Deyang City Deyang China; ^2^ Department of Respiratory and Critical Medicine People’s Hospital of Deyang City Deyang China; ^3^ Department of Hepatobiliary Pancreatic Surgery People’s Hospital of Deyang City Deyang China; ^4^ Department of Neurology People’s Hospital of Deyang City Deyang China

**Keywords:** glioblastoma, invasion, lncRNA GAS8‐AS1, lncRNA NEAT1, proliferation

## Abstract

**Background:**

LncRNA GAS8‐AS1 has been reported to participate in several types of cancer, while its role in glioblastoma (GBM) is unknown. In the present study, we aimed to investigate the function of GAS8‐AS1 in GBM and the underlying mechanisms.

**Methods:**

The expression levels of GAS8‐AS1 and NEAT1 in GBM patients and the healthy controls were measured by performing RT‐qPCR. Diagnostic values of plasma GAS8‐AS1 and NEAT1 for GBM were analyzed by performing ROC curve analysis with GBM patients as true positive cases and the healthy controls as true negative cases. Linear regression analysis was performed to study the correlation between the expression levels of GAS8‐AS1 and NEAT1. The expression levels of GAS8‐AS1 and NEAT1 in GBM cells were also determined by RT‐qPCR. CCK‐8 and transwell invasion assays were performed to detect the proliferation and invasion of GBM cells. Western blot assay was performed to detect the expression levels of β‐catenin, Axin2, c‐myc, cyclin D1, and GAPDH in GBM cells.

**Results:**

GAS8‐AS1 was downregulated, while lncRNA NEAT1 was upregulated in the plasma of GBM patients. Altered expression levels of GAS8‐AS1 and NEAT1 distinguished GBM patients from the healthy controls. The expression of GAS8‐AS1 and NEAT1 was inversely correlated only in GBM patients. Overexpression of GAS8‐AS1 reduced the expression levels of NEAT1 in GBM cells, while knock‐down of GAS8‐AS1 increased the expression levels of NEAT1. However, overexpression of NEAT1 showed no significant effects on the expression of GAS8‐AS1. Knock‐down of GAS8‐AS1 promoted GBM cell proliferation and invasion and enhanced the activation of the Wnt/β‐catenin pathway. However, the effects of knock‐down of GAS8‐AS1 were alleviated by the knock‐down of NEAT1.

**Conclusion:**

Overexpression of GAS8‐AS1 inhibits GBM cell proliferation and invasion by downregulating NEAT1.

## Graphical abstract

GAS8‐AS1 was downregulated in GBM and GAS8‐AS1 overexpression may inhibit GBM cell proliferation by downregulating NEAT1.

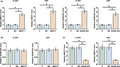

## 1 INTRODUCTION

Glioblastoma (GBM) is the most common type of brain tumor (De Barros et al., 2019). GBM affects 3–4 patients out of 100,000 people (Siegel et al., 2020). In spite of the low incidence rate, GBM is considered as a major cause of cancer‐related deaths due to its aggressive nature (Siegel et al., 2020). In effect, only less than 3% of patients with GBM can live longer than 5 years after the initial diagnosis (Diwanji et al., 2017; Zhang et al., 2015). In addition, incidence of GBM in recent years showed an increasing trend (Siegel et al., 2020). The unclear molecular pathogenesis is the major challenge in the prevention and clinical treatment of GBM (Alifieris & Trafalis, 2015). Therefore, investigating the molecular pathways involved in GBM and exploring novel therapeutic targets are still critical.

Previous studies have shown that genetic alterations are closely correlated with the initiation, development, and progression of GBM (Agnihotri et al., 2013; Li et al., 2016; Liu et al., 2016). However, the oncogenes and tumor suppressors identified so far are not sufficient to cover the complex molecular pathogenesis of GBM. Long (>200 nt) noncoding RNAs (lncRNAs) are involved in the development of many cancers, such as GBM, by regulating the expression of cancer‐related genes (Gao et al., 2019; Liu et al., 2020). For instance, lncRNA RMST enhances FUS SUMOylation to suppressed GBM Cell mitophagy (Liu et al., 2020), and lncRNA MNX1‐AS1 inhibits the expression of miR‐4443 to promote GBM progression (Gao et al., 2019). However, functions of most lncRNAs in GBM are still unclear. Therefore, studies on the involvement of lncRNAs in cancer biology are always needed.

LncRNA GAS8‐AS1 has been reported to participate in several types of cancer, such as papillary thyroid carcinoma (Pan et al., 2016; Zhang et al., 2017). However, its role in GBM is elusive. Our preliminary RNA‐seq data revealed the downregulation of GAS8‐AS1 in GBM and its inverse correlation with NEAT1, a lncRNA promoting GBM progression by activating the Wnt/β‐catenin pathway (Chen et al., 2018). In the present study, we further investigated the possible role of GAS8‐AS1 in GBM and whether if functions by regulating NEAT1.

## 2 MATERIALS AND METHODS

### 2.1 Research subjects

This study included 51 GBM patients (33 males and 18 females, 39 to 68 years old, mean age 54.4 ± 6.7 years old) selected from 78 GBM patients admitted at People's Hospital of Deyang City from May 2015 to May 2018. Inclusion criteria were as follows: 1) GBM patients with no history of previous malignancy; 2) complete medical records; 3) first diagnosis and no therapies were received before admission. Exclusion criteria were as follows: 1) complicated with other medical disorders; 2) patients transferred from other hospitals. GBM in all cases was located in the supratentorial region. During the same time period, 51 healthy volunteers (33 males and 18 females, 38 to 69 years old, mean age 55.1 ± 6.5 years old) were also included to serve as the control group. All healthy volunteers received systemic physiological examinations in aforementioned hospital, and all physiological parameters were within normal range. All patients and healthy volunteers signed the informed consent. This study was approved by the Ethics Committee of People's Hospital of Deyang City.

### 2.2 Plasma and cell lines

Before therapies, blood (5 ml) was extracted from each patient and the healthy controls under fasting conditions. Blood was centrifuged at 1,200 g for 15 min in EDTA tubes to prepare plasma samples. Human GBM cell lines U‐251 (Sigma‐Aldrich) and U87 (ATCC) were used to perform in vitro cell experiments. All cells were cultivated following the manufacturer's instructions. DMEM (10% FBS) was used as the culture medium, and cells were cultured at 37 ºC with 5% CO_2_.

### 2.3 RT‐qPCR

Total RNAs were extracted from plasma and cells of U‐251 and U87 cell lines using Ribozol RNA Extraction Reagent (Thomas Scientific). Invitrogen™ AMV Reverse Transcriptase (Fisher Scientific) was used to perform reverse transcriptions to obtain cDNAs. The qPCR reaction systems were prepared using KAPA SYBR® FAST Universal Kit (Sigma‐Aldrich). The expression levels of GAS8‐AS1 and NEAT1 were measured with 18S rRNA as endogenous control. All PCR reactions were performed in triplicate manner. The expression level of GAS8‐AS1 (or NEAT1) in experimental groups was first normalized to 18S rRNA level using 2^‐ΔΔCT^ method and then was further normalized to the relative level of GAS8‐AS1 (or NEAT1) in the control group.

### 2.4 Cell transient transfections

The pcDNA3.1 vectors expressing GAS8‐AS1 or NEAT1 were constructed by Sangon. The GAS8‐AS1 and NEAT1 siRNAs, and negative control (NC) were obtained from Genechem. All cell transfections were performed using Lipofectamine 2000 reagent (Thermo Fisher Scientific) with 10 nM vectors. Cells without transfections were used as the control, and cells transfected with empty vectors were used as the negative control. All subsequent experiments were performed at 36 hr after cell transfection.

### 2.5 Cell proliferation assay

Cells of U‐251 and U87 cell lines were collected at 36 hr after transfection to perform in vitro cell proliferation assays. Briefly, cells were mixed with DMEM (10% FBS) to prepare single cell suspensions. Cell density was adjusted to 3 × 10^4^ cells per well. Cell suspensions were transferred to a 96‐well cell culture plate with 0.1 ml per ml. Cells were cultivated at 37°C with 5% CO_2_. CCK‐8 (10 μl, Sigma‐Aldrich) was added into each well every 24 hr until 96 hr after the beginning of cell culture. After that, cells were cultivated for another 4 hr. Finally, OD values at 450 nM were measured to represent cell proliferation.

### 2.6 Transwell invasion assay

Transwell inserts were pretreated with 50 μl matrigel (dissolved in 300 μl RPMI‐1640 medium) at 37℃ overnight. Then, 1 × 10^5^ GBM cells were plated into the upper chamber of transwell inserts coated with matrigel, and 100 μl RPMI‐1640 medium containing 10% FBS was added into the lower chamber of transwell inserts. After incubation for 24 hr, the noninvasive cells were cleaned using a cotton swab, and the invasive cells were fixed by 4% paraformaldehyde and stained by eosin. The number of invasive cells from three randomly selected fields was counted.

### 2.7 Western blot assay

Total proteins were extracted from GBM cells using RIPA lysis buffer. Protein samples were quantified using a bicinchoninic acid protein assay kit (Beyotime). Equal amount of total protein was separated by SDS‐PAGE and then transfected onto a PVDF membrane. After incubation with 5% skim milk overnight, the membrane was incubated with antibodies against β‐catenin (1:500; Abcam), Axin2 (1:500; Abcam), c‐myc (1:1,000; Abcam), cyclin D1 (1:1,000; Abcam), and GAPDH (1:1,000; Abcam), followed by incubation with HRP‐conjugated secondary antibody (1:1,000; Beyotime). Finally, the target proteins were visualized with a ECL‐HRP detection kit (Millipore).

### 2.8 Statistical analyses

Three biological replicates were included in each experiment. Differences between patients and the healthy controls were analyzed by unpaired *t* test. Differences among different cell transfection groups were analyzed by one‐way ANOVA and Tukey's test. Diagnostic values of plasma GAS8‐AS1 and NEAT1 for GBM were analyzed by performing ROC curve analysis with GBM patients as true positive cases and healthy controls as true negative cases. Areas under the curves (AUCs) were used to evaluate the diagnostic value of each marker: AUC value > 0.9, excellent diagnostic efficacy; 0.7 < AUC value ≤ 0.9, good diagnostic efficacy; 0.5 < AUC value ≤ 0.7, poor diagnostic efficacy; AUC < 0.5, no diagnostic value. Linear regression analysis was performed to study the correlation between the expression levels of GAS8‐AS1 and NEAT1. Differences were considered significant with *p* <.05.

### 2.9 Ethical approval

The present study was approved by the Ethics Committee of People's hospital of Deyang City. The research has been carried out in accordance with the World Medical Association Declaration of Helsinki. All patients and healthy volunteers provided written informed consent prior to their inclusion within the study.

## 3 RESULTS

### 3.1 The expression levels of GAS8‐AS1and NEAT1 were altered in GBM patients compared to that in healthy controls

**FIGURE 1:**
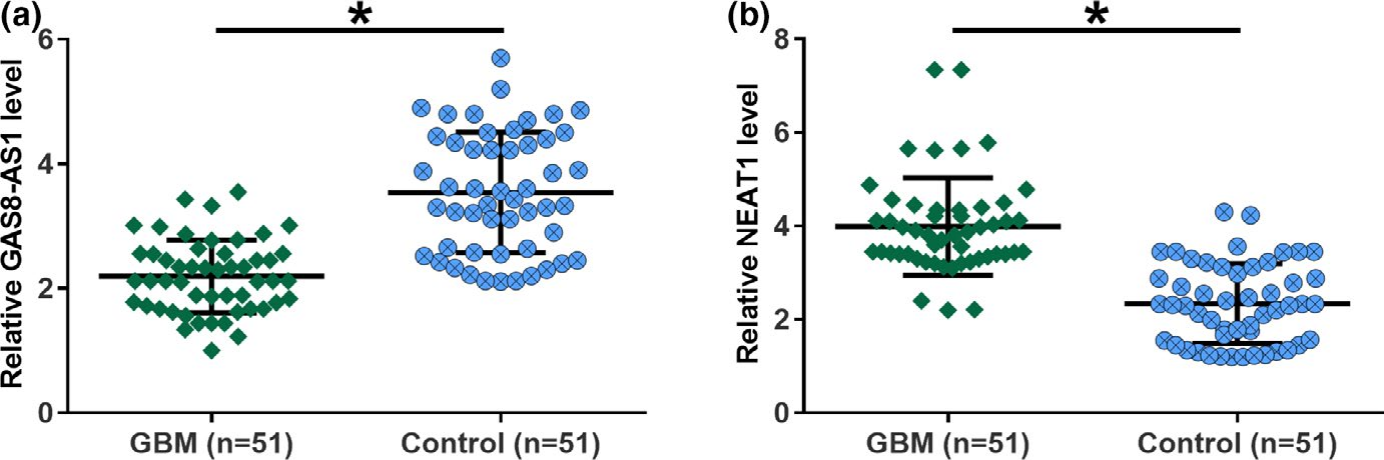
The expression levels of GAS8‐AS1and NEAT1 were altered in GBM patients compared to healthy controls. Analysis of the expression data of GAS8‐AS1 and NEAT1 by unpaired *t* test showed that GAS8‐AS1 was significantly downregulated (a), while lncRNA NEAT1 was significantly upregulated (b) in GBM patients than that in healthy controls (*, *p* <.05). *N* = 51

The expression levels of GAS8‐AS1 and NEAT1 in both GBM patients and healthy controls were measured by RT‐qPCR. The expression data were analyzed by unpaired *t* test. It was observed that GAS8‐AS1 was significantly downregulated in GBM patients than that in healthy controls (Figure 1a, *p* < .05). However, NEAT1 was upregulated in GBM patients than that in in healthy controls (Figure 1b, *p* < .05).

### 3.2 Altered expression levels of GAS8‐AS1 and NEAT1 showed diagnostic potentials for GBM

**FIGURE 2:**
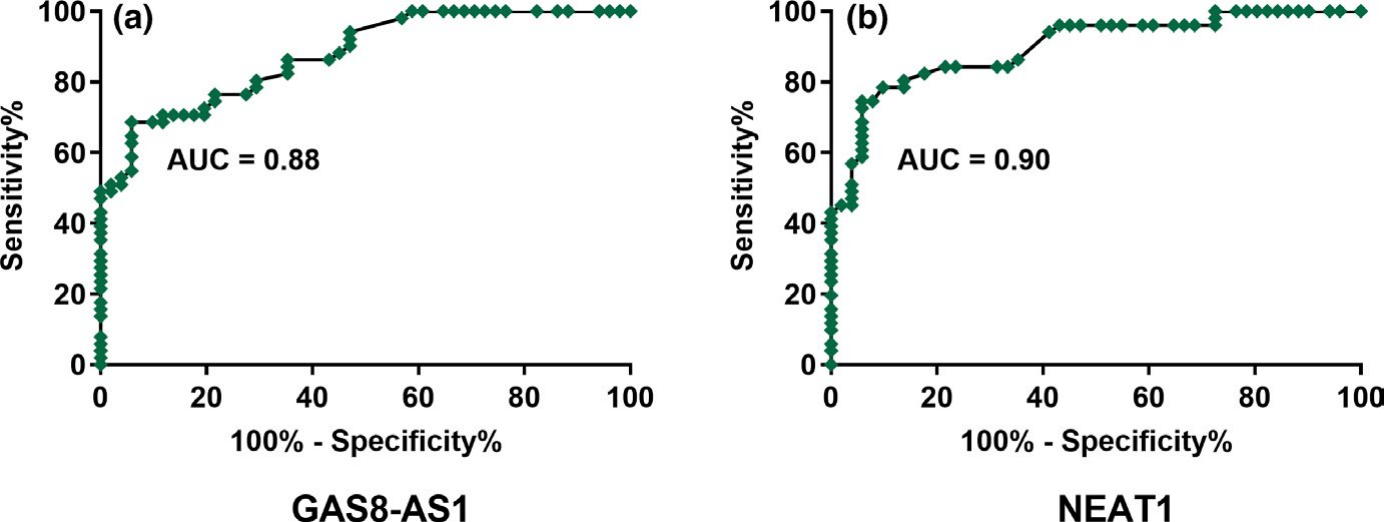
Altered expression levels of GAS8‐AS1 and NEAT1 showed diagnostic potentials for GBM. ROC curve analysis showed that altered expression levels of GAS8‐AS1 (a) and NEAT1 (b) distinguished GBM patients from the healthy controls. *N* = 51

Diagnostic values of the expression of GAS8‐AS1 and NEAT1 for GBM were analyzed by performing ROC curve analysis with GBM patients as true positive cases and healthy controls as true negative cases (Figure 2). For plasma GAS8‐AS1, area under the curve (AUC) was 0.88 (standard error: 0.033; 95% confidence interval: 0.81–0.94; *p* <.001). For plasma NEAT1, AUC was 0.90 (standard error: 0.031; 95% confidence interval: 0.84–0.96; *p* <.001). The results suggest that GAS8‐AS1 and NEAT1 may be good diagnostic values for GBM.

### 3.3 GAS8‐AS1 and NEAT1 were inversely correlated in GBM

**FIGURE 3:**
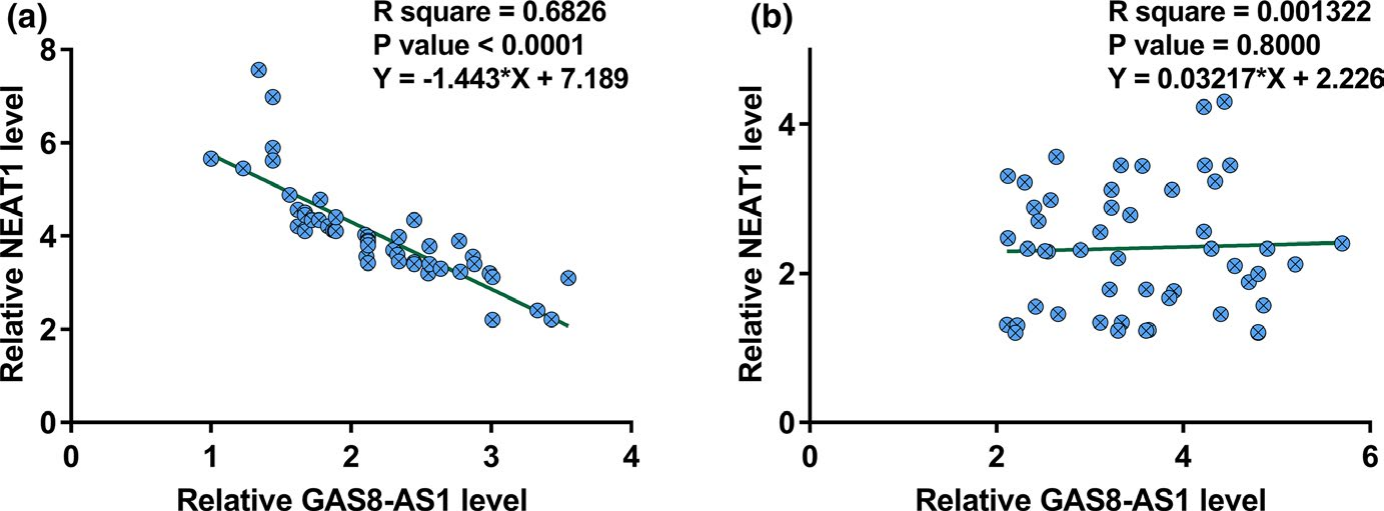
GAS8‐AS1 and NEAT1 were inversely correlated in GBM. Linear regression analysis showed that GAS8‐AS1 and NEAT1 were inversely correlated in GBM (a), but not in healthy controls (b). *N* = 51

Linear regression analysis was performed to study the correlation between the expression levels of GAS8‐AS1 and NEAT1. It was observed that the expression of GAS8‐AS1 and NEAT1 was inversely correlated in GBM patients (Figure 3a). In contrast, the correlation between the expression levels of GAS8‐AS1 and NEAT1 was not significant in healthy controls (Figure 3b).

### 3.4 GAS8‐AS1 may be an upstream inhibitor of NEAT1 in GBM

**FIGURE 4:**
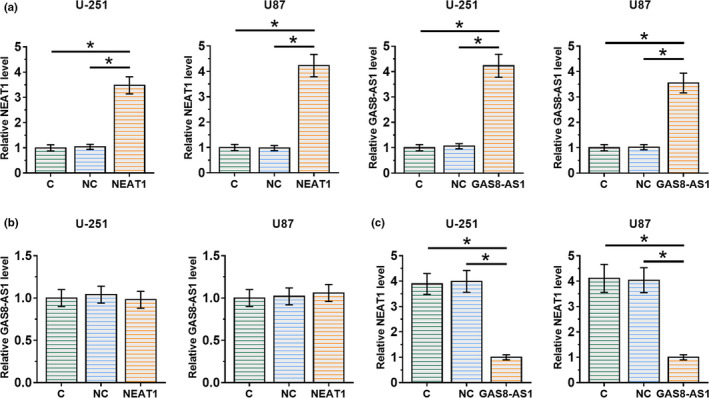
GAS8‐AS1 may be an upstream inhibitor of NEAT1. Compared to the two controls, including control (C) and negative control (NC), the expression levels of GAS8‐AS1 and NEAT1 were significantly increased at 36 hr after transfection (a). Compared to the two controls (b), overexpression of NEAT1 showed no significant effects on GAS8‐AS1, while overexpression of GAS8‐AS1 downregulated NEAT1 in GBM cells (c) (*, *p* <.05). *N* = 3

GAS8‐AS1 and NEAT1 expression vectors were transfected into U‐251 and U87 cells. Compared to the control (C) and negative control (NC), the expression levels of GAS8‐AS1 and NEAT1 were significantly increased at 36 hr after transfection (Figure 4a, *p* < .05). Compared to the two controls (Figure 4b), overexpression of NEAT1 showed no effects on the expression of GAS8‐AS1, while overexpression of GAS8‐AS1 downregulated NEAT1 GBM cell lines (Figure 4c , *p* < .05). Moreover, knock‐down of GAS8‐AS1 obviously increased the expression levels of NEAT1, while knock‐down of NEAT1 had no effect on the expression of GAS8‐AS1 in U‐251 and U87 cells (Figure S1).

### 3.5 GAS8‐AS1 regulated proliferation, invasion, and activation of Wnt/β‐catenin pathway in GBM cells through NEAT1

**FIGURE 5:**
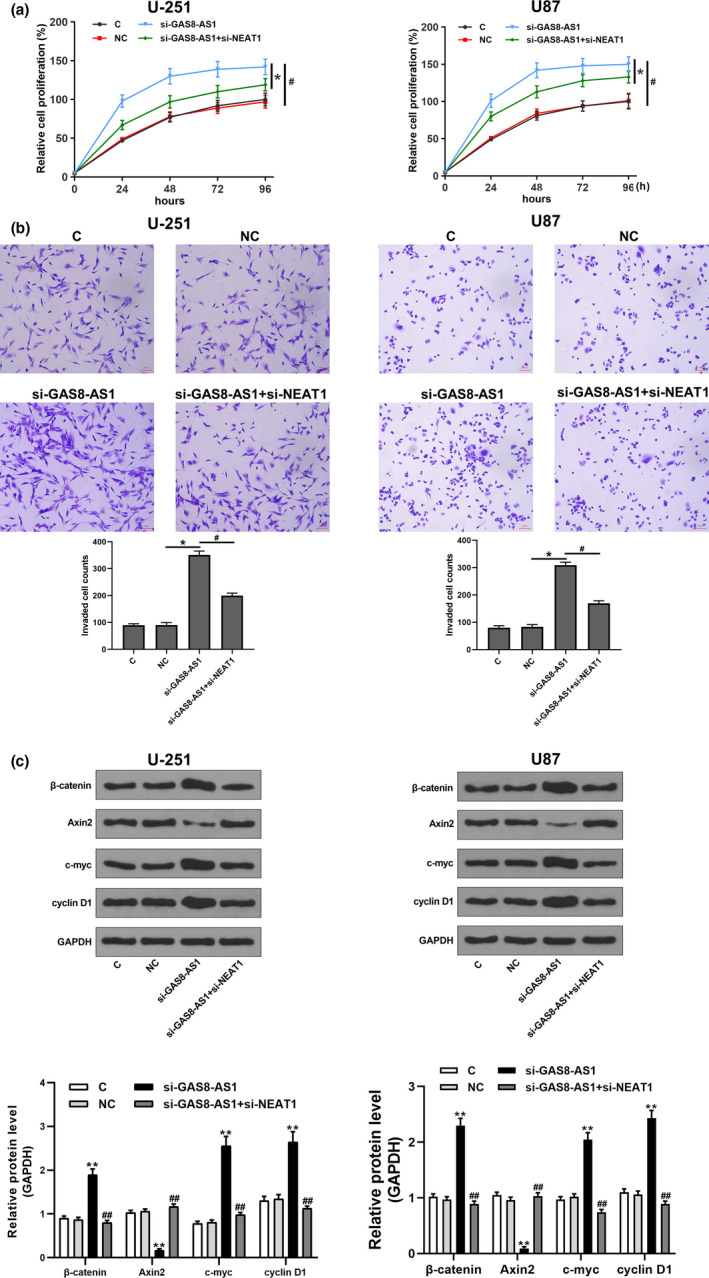
GAS8‐AS1 regulated GBM cell proliferation, invasion, and activation of Wnt/β‐catenin pathway in GBM cells through NEAT1. Compared to the two controls, including control (C) and negative control (NC), knock‐down of GAS8‐AS1 significantly promoted cell proliferation (a) and invasion (b) and enhanced the activation of the Wnt/β‐catenin pathway (c), while knock‐down of NEAT1 alleviated the effects of knock‐down of GAS8‐AS1 (*, *p* <.05 and **p <.01 versus NC; #*p* <.05 and ##*p* <.01 versus si‐GAS8‐AS1). *N* = 3

Comparing to the control (C) and negative control (NC), knock‐down of GAS8‐AS1 remarkably promoted GBM cell proliferation and invasion, while knock‐down of NEAT1 attenuated the effects of knock‐down of GAS8‐AS1 on proliferation and invasion (Figure 5a and b). In addition, knock‐down of GAS8‐AS1 significantly increased the expression levels of β‐catenin, c‐myc, and cyclin D1, while reduced the expression levels of Axin2 protein (Figure 5c). It suggested that knock‐down of GAS8‐AS1 promoted the activation of the Wnt/β‐catenin pathway. However, the effect of knock‐down of GAS8‐AS1 on the expression of these proteins was alleviated by the knock‐down of NEAT1 (Figure 5c), suggesting that NEAT1 was involved in GAS8‐AS1‐mediate regulation of the Wnt/β‐catenin pathway.

## 4 DISCUSSION

GAS8‐AS1 is downregulated in papillary thyroid carcinoma, indicating its tumor suppressive role in this disease. Our study explored the involvement of GAS8‐AS1 in GBM. The U251 and U87 cell lines are commonly used as experimental models of glioblastoma. However, a recent study has reported that the differences in nicotinamide nucleotide metabolic process regulation, RNA splicing, glycolysis, and purine metabolism exist between the U87 and U251 cell lines (Li et al., 2017). Therefore, in the study, these two cell lines were both used to ensure the consistency of experimental results. Here, we found that GAS8‐AS1 was downregulated in GBM. Moreover, downregulation of GAS8‐AS1 promoted proliferation and invasion and enhanced the activation of the Wnt/β‐catenin pathway in GBM cells by regulating NEAT1.

Previous studies have shown that the development and progression of GBM are accompanied by altered expression of a huge number of lncRNAs (Han et al., 2012). Differentially expressed lncRNAs have been demonstrated as critical players in GBM (Liu et al., 2018; Yao et al., 2015). LncRNA XIST was downregulated in GBM, and overexpression of XIST resulted in inhibited progression of GBM through the upregulation of tumor suppressive miR‐152 (Yao et al., 2015). In contrast, lncRNA LINC00152 was upregulated in GBM and promoted the development of GBM by regulating miR‐107 (Liu et al., 2018). LncRNAs participate in cancer biology mainly by indirectly affecting cancer cell behaviors (Gao et al., 2019; Liu et al., 2020). LncRNA GAS8‐AS1 has been reported to be a tumor suppressor in papillary thyroid carcinoma, colorectal cancer, and osteosarcoma (Pan et al., 2016; Schmitt & Chang, 2016; Zha et al., 2020; Zhao et al., 2019). And GAS8‐AS1 inhibits tumorigenesis by repressing cancer cell proliferation, migration, and invasion (Schmitt & Chang, 2016; Zha et al., 2020; Zhao et al., 2019). To the best of our knowledge, we first uncovered that GAS8‐AS1 was downregulated in GBM, and it had an inhibitory effect on GBM cell proliferation and invasion (Zhao et al., 2019).

Altered expression of lncRNAs may result in changes in the expression levels of certain oncogenes or tumor suppressors. Therefore, lncRNAs may play their roles in cancer development by affecting the synthesis of protein products. In addition, lncRNA can also affect other noncoding RNAs, such as miR‐152 and miR‐107, to inhibit or promote cancer development (Liu et al., 2018; Yao et al., 2015). An increasing number of studies have focused on the interactions between lncRNAs and lncRNAs in the progress of cancer. For example, lncRNA DANCR was found to promote gastric cancer development by inhibiting lncRNA LET. LncRNA GAS8‐AS was found to suppress colorectal cancer cell proliferation by reducing the expression levels of lncRNA AFAP1‐AS1 (Zhao et al., 2019). Here, we revealed that GAS8‐AS1 inhibited GBM cell proliferation and invasion by downregulating lncRNA NEAT1. Further studies are needed to explore the possible mechanisms by which GAS8‐AS1 regulates NEAT1.

## 5 CONCLUSIONS

In conclusion, downregulation of GAS8‐AS1 in GBM promotes cell proliferation and invasion and enhances the activation of the Wnt/β‐catenin pathway by upregulating NEAT1.

## CONFLICT OF INTEREST

The authors declare that they have no competing interests.

## AUTHOR CONTRIBUTIONS

All authors contributed to data analysis, drafting or revising the article, gave final approval of the version to be published, and agree to be accountable for all aspects of the work.

## CONSENT FOR PUBLICATION

Not applicable.

### PEER REVIEW

The peer review history for this article is available at https://publons.com/publon/10.1002/brb3.2128.

## Supporting information

Figure S1

## Data Availability

The analyzed data sets generated during the study are available from the corresponding author on reasonable request.
