# Supplementary figures and images for: LncRNA GAS8‐AS1 downregulates lncRNA NEAT1 to inhibit glioblastoma cell proliferation
Source: Brain Behav. 2021 May 4;11(6):e02128. doi: 10.1002/brb3.2128 (PMC8213648; doi:10.1002/brb3.2128)

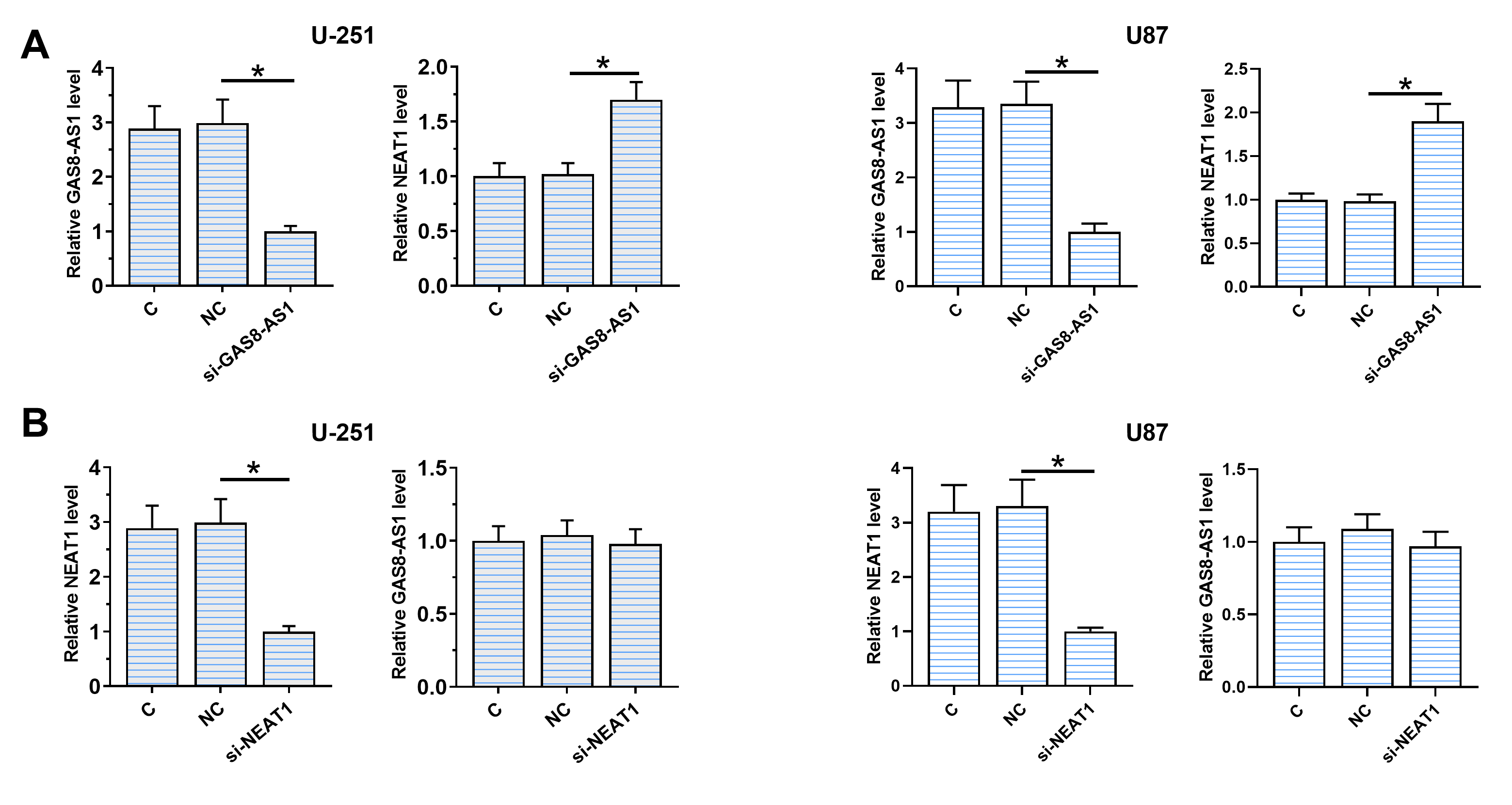

Supplement: Supplementary file 1 — Figure S1 [file BRB3-11-e02128-s001.tif]
